# Amp-C*Klebsiella*-Induced Chorioamnionitis and Associated Abnormalities in Cardiotocography: A Case Report with a Narrative Review

**DOI:** 10.1155/2022/7127236

**Published:** 2022-11-23

**Authors:** Sufia Athar, Anvar P. Vellamgot, Lolwa Mohammed Alansari

**Affiliations:** ^1^Department of Obstetrics and Gynecology, Al Wakra Hospital, HMC, Al Wakra, Qatar; ^2^Department of Neonatology, Al Wakra Hospital, HMC, Al Wakra, Qatar

## Abstract

**Introduction:**

Infections caused by multidrug-resistant organisms are on the rise in obstetric patients. Chorioamnionitis is associated with adverse pregnancy outcomes. If caused by multidrug-resistant organisms, chorioamnionitis is associated with high maternal and fetal morbidity. Due to the paucity of the literature and the challenges associated with their diagnosis, the diagnosis is usually delayed. This often leads to delays in management, and hence, adverse maternal and neonatal outcomes are noted. *Important Clinical Findings*. The patient presented with prelabour rupture of membranes for three days. She developed chorioamnionitis in labour, which was refractory to broad spectrum antibiotics. Persistent tachycardia with variable decelerations followed by prolonged fetal deceleration was observed in cardiotocography. Delivery of baby was done by cesarean section in the view of pathological findings in cardiotocography. *The primary diagnoses*, *interventions*, *and outcomes*. Based on the placental culture results, Amp-C* Klebsiella*-induced chorioamnionitis was diagnosed. Ertapenem was commenced after the sensitivity results. Good feto-maternal outcomes were observed. The neonate was admitted to the neonatal intensive care unit and discharged home in stable condition.

**Conclusions:**

Cases of multidrug-resistant organisms-induced chorioamnionitis are rarely reported, though they are associated with maternal morbidity and poor neonatal outcome. In cases of chorioamnionitis, caution should be taken in cases that are refractory to broad-spectrum antibiotics, and multidrug-resistant organisms should be suspected and managed to improve pregnancy outcome.

## 1. Introduction

With the augmenting rise in the prevalence of multiple drug-resistant organisms (MDRO) in healthcare worldwide, there is a proportionate increase in their prevalence in obstetrics patients [1, 2]. Most common organisms are the antibiotic-resistant Gram-negative bacteria (*Enterobacteriaceae*) which produce extended-spectrum-lactamases (ESBLs), metallo *β*-lactamases, carbapenemases, and Amp-C-lactamases [2–4]. However, the diagnosis and management of these cases are challenging due to limited studies and antibiotic resistance.

In women with chorioamnionitis (CA) caused by MDRO, the diagnosis is often delayed due to atypical presentation and is associated with poor neonatal outcomes [5]. Once the diagnosis of CA is established, usually, broad-spectrum antibiotics are commenced. However, due to antibiotic resistance in cases of CA caused by MDRO, maternal and neonatal morbidity is unexpectedly higher compared to usual CA cases [5]. In a review by Ballot et al. (2019), a statistically significant rise in MRDO isolates from neonates was reported (0.39 in 2013 vs. 1.4 in 2015). Maternal and neonatal outcomes are usually poor in these cases [5, 6]. The prevalence of MDRO in neonates was reported as 8.8%, and mortality rate in these neonates was 33.3% [6]. It was also noted that the neonates with MDRO CA needed resuscitation at birth and 45% required oxygen therapy for four weeks and more [6]. These studies give an insight into the magnitude of the MDRO-induced neonatal sepsis cases and associated complications. We present an interesting case of Amp-C producing *Klebsiella pneumoniae*-induced CA and the challenges in the diagnosis, management, and associated fetal morbidity.

## 2. Case Report

A 31-year-old primigravida woman presented at 39 weeks of pregnancy to the Obstetric Emergency with complaints of vaginal leaking for three days and labour pains for 4 hours. She had insignificant medical, family, and psychosocial history. The vaginal leaking was minimal initially, so she did not report it earlier. She was having regular contractions for the last 3 hours. There was no history of fever or abnormal vaginal discharge. She had regular antenatal visits during her pregnancy from the first trimester. Her antenatal period was uneventful, except for an incidentally diagnosed ovarian dermoid cyst in her nuchal translucency ultrasound. After this, regular follow-up ultrasounds were performed, and the size of the ovarian cyst did not show any change. She did not have pain or other symptoms with the ovarian cyst, so she was offered conservative management and planned to do surgical intervention after her delivery.

On examination, her vital signs were stable (pulse 80/m, T: oral 36.8°C, RR: 20/m, BP: 127/66 mm Hg SpO2: 100%). The fundal height corresponded to her period of gestation. Speculum examination did not reveal any liquor, but a heavy show was noted. On vaginal examination, the cervix was very soft, 1.5 cm dilated, 80% effaced, absent membrane, and the vertex was at -3 station. Antibiotic prophylaxis for Group B streptococci was commenced because of rupture of membranes (ROM) for more than 72 hours. Laboratory findings demonstrated high absolute neutrophil count (ANC) of 10.7 × 10^3/*μ*L (normal range 2–7 × 10^3/*μ*L) and C-reactive protein of 53 mg/L. The admission hemoglobin level was 12.1 g/dl, and the white blood cell count (WBC) was 13.7 10^3/microliter. Her admission cardiotocography (CTG) revealed 2 variable decelerations for 2–3 minutes. The rest of the CTG parameters were normal (baseline 140 bpm, variability 5–10 bpm, accelerations were noted, and the fetus was reactive). The CTG improved after internal manoeuvres. She was transferred to the labour room for labour augmentation.

**Figure 1:**
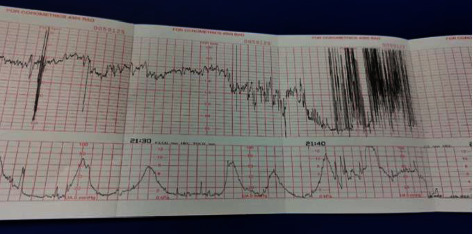
CTG of the patient before shifting for emergency cesarean section.

Labour was augmented, and CTG remained normal. She had regular contractions after 2 hours of augmentation. In the repeat vaginal examination after 4 hours of regular contraction, the cervix was 4 cm dilated, 100% effaced, the membrane was absent, the vertex was at -3 station, and thick meconium was noted. After 2 hours, she developed fever (38 C) with a maternal pulse of 140 bpm and fetal tachycardia (baseline of 170 bpm). Diagnosis of clinical chorioamnionitis was made and broad-spectrum antibiotics were started. Complete blood count and C-reactive protein (CRP) were sent. Lab results showed an increase in WBC, ANC, and CRP (WBC- 18.9 × 10^3/Ul, ANC-17.0 × 10^3/Ul, and CRP of 53 mg/L). Soon she had prolonged deceleration for 6 minutes, followed by recurrent variable decelerations. Internal manoeuvres were performed, and improvement was noted in CTG. After an hour she had another prolonged deceleration for 6–8 minutes which did not settle with any interventions and she was shifted for emergency cesarean (Figure 1).

She had uncomplicated cesarean delivery, and the placenta was sent for culture. She delivered a boy weighing 3.42 kg and APGAR scores of 9 and 10 at the first and fifth minute of birth, respectively. Arterial pH was 7.133 with a base excess of −5.8 mmol/L and venous pH was 7.20 and the base excess was −8.1 mmol/L. Thick meconium was observed during the cesarean section. In the postoperative period, she had spikes of fever more than 39 C, despite being on broad-spectrum antibiotics. Placental culture developed growth of Amp-C beta-lactamase-producing* Klebsiella pneumoniae*. Ertapenem was commenced after the sensitivity results (Table 1). Within 24 hours, she was afebrile. The postoperative period was uneventful and she was discharged home on the third postoperative day, and antibiotics were continued for another week.

Soon after delivery, the baby developed respiratory distress and required continuous positive airway pressure (CPAP) of 5 cm water with an inspired oxygen concentration (FiO2) of 30%. He was admitted to the neonatal intensive care unit (NICU) on CPAP. The initial capillary blood gas (CBG) was normal. The chest X-ray showed evidence of pneumonia and minimal right-sided pneumothorax. He was started on intravenous ampicillin and amikacin. By 14 hours of age, he was intubated due to increasing distress and FiO2 requirement of up to 50% to maintain saturation >95%. He also received 100 mg/kg of bovine surfactant. His clinical status and ventilation requirements improved gradually and were extubated to a high-flow nasal cannula by 44 hours of age. After extubation, he continued to need Fio2 of 35%. The bedside ECHO showed evidence of mild pulmonary hypertension. His blood culture was sterile, white blood cell count was normal, and C-reactive protein was 15 mg% and 12 mg% at 48 hours intervals. Because of the continued oxygen requirement, X-ray evidence of pneumonia, and the positive maternal blood culture, intravenous antibiotics were continued for ten days. Nasal oxygen was discontinued by 12 days and the baby was discharged home by 14 days of age.

## 3. Discussion and Review of the Literature

In the last two decades, a significant increase in MDRO cases has been recorded. These organisms produce enzymes that cause resistance to even the most potent broad-spectrum antibiotics. These enzymes were initially noted in the *Enterobacteriaceae* family, but in the last few decades, these were detected in other species like *Pseudomonas aeruginosa*, *Haemophilus influenzae*, and *Neisseria gonorrhoeae* [2]. The plasmid-mediated Ambler class C beta-lactamases (Amp-C*β*-lactamases) were noted in *Escherichia coli* and *Klebsiella pneumoniae* [3, 4]. They are not only resistant to antibiotics but also needed a sophisticated methodology for detection. It often leads to delays in detection or missed diagnosis [7]. The polymerase chain reaction (PCR) remains the gold standard for the diagnosis of Amp-C producers [8]. Carbapenems are the drug of choice for treating these organisms as they are resistant to cephalothin, cefazolin, cefoxitin, most penicillins, and *β*-lactamase inhibitor-*β*-lactam combinations [8].

Cases of CA are usually diagnosed based on clinical criteria [9, 10]. In women with clinical chorioamnionitis with MDRO, symptoms may be masked, attributing to delays in the diagnosis and poor neonatal outcome [1].

*Klebsiella pneumoniae*, an opportunistic pathogen, is a leading cause of neonatal sepsis, especially in low- and middle-income countries [11]. However, reports regarding in-utero infection due to *K*. *pneumoniae* are extremely scarce [12]. A few cases of *Klebsiella pneumonia* chorioamnionitis resulting in fetal demise have been reported [13, 14]. Neonatal *Klebsiella* sepsis is associated with high (up to 20%) mortality (Mai 2010) [15]. *Klebsiella pneumoniae* is notorious for its ability to acquire antibiotic resistance determinants, and it belongs to the “critical” category in the WHO global priority pathogen list [16]. In a study done in Taiwan, 376 episodes of Gram-negative bacteremia (GNB) were analyzed [17]. Underlying neurologic sequelae (22.9% vs. 13.4%), renal disease (12.9% vs. 1.3%), previous episode of bacteremia (35.7% vs. 23.5%), use of total parenteral nutrition (80% vs. 67.6%), and use of a central venous catheter (87.1% vs. 73.2%) were significantly high in MDR GNB as compared to non-MDR GNB cohort. The MDRO-induced neonatal sepsis is associated with a higher need for mechanical ventilation and resuscitation at birth 6. These infections were higher in low birth weight babies and preterm babies [6]. Neonatal sepsis with MDRO is associated with significantly higher mortality [18, 19].

In our case, the patient presented with prolonged rupture of membranes (>72 hours) which is an identifiable risk factor for CA [20]. As penicillin injections were given from the admission, the clinical symptoms of CA were masked till she developed septicemia. This may be attributable to the delay in the diagnosis of the sepsis, and hence, it was associated with neonatal morbidity after delivery. Fortunately, with the first spike in temperature, broad-spectrum antibiotics were commenced, which led to a good maternal outcome. In a study by Shittu et al., poor maternal morbidity was noted in women with MDRO CA leading to maternal septicemia and wound infection. In their study, the patient had readmission with the same MDRO-induced surgical site infection which caused CA [5].

In our case, CTG abnormalities were noted even before the clinical diagnosis of CA. Admission CTG was suspicious and unprovoked decelerations were noted. CTG resumed a normal pattern after intravenous antibiotics and internal manoeuvres. However, once she developed fever, the CTG pattern was abnormal with baseline tachycardia, poor variability, and variable decelerations. Later she developed prolonged decelerations for which she had an emergency cesarean section. Thick meconium during the cesarean suggested fetal distress.

In the literature, no correlation between a low arterial pH (<7.20) and fetal heart rate patterns was noted in women with CA if neonates were delivered in less than twelve hours since the diagnosis of CA was made [21]. On the other hand, the absence of a cycling pattern and maternal tachycardia were associated with poor neonatal outcome [22]. In another case report, recurrent variable decelerations were noted with baseline tachycardia and poor variability with MDRO-induced CA [5]. Sukumaran et al. reported an increase in baseline fetal heart rate and variable decelerations in women with CA [23]. There is hardly any literature on the CTG patterns in CA with MDRO cases. As MDRO, cases are associated with neonatal morbidity, CTG abnormalities are quite possible. CTG interpretation and management plans in these cases should be done with caution.

Cases of MDRO-induced CA are rarely reported, though they are associated with maternal morbidity and poor neonatal outcome. In women on antibiotic prophylaxis in labour, the symptoms may be masked leading to the delayed diagnosis of CA. Due to multiple drug resistance, the management of CA in these cases may be delayed. Hence, in cases of CA, caution should be taken in cases who are refractory to broad-spectrum antibiotics and MDRO should be suspected. In these cases, labour should be augmented to expedite the delivery. In cases where the diagnosis is suspected in early labour, cesarean may be considered. However, larger studies are needed in this regard to draw any conclusions.

## 4. Conclusions

Chorioamnionitis remains a diagnostic challenge for clinicians as symptoms of CA may be influenced by other infections, epidural analgesia, and maternal factors. If CA is caused by MDRO, diagnostic challenges are further intricated due to no response to conventional antibiotics and associated cardiotocography abnormalities. Placental cultures sent after delivery usually take 24–72 hours for the final interpretation of bacterial load and antibiotic sensitivity. In these cases, it is desirable to consider changing antibiotics sensitive to MDRO and hastening delivery to prevent maternal and fetal morbidity and mortality. Studies and reviews in this regard will be helpful to establish recommendations for the management of these cases.

### 4.1. Strengths

This case report provides a detailed description of MDRO-induced CA, which may aid in understanding the diagnostic and management issues in these cases. Our case study also gives an insight into suspecting MDRO in cases that are refractory to antibiotics given for CA. It would reduce maternal and neonatal morbidity if we aptly intervene.

### 4.2. Limitations

Subjectivity in presentation and evaluation of the case study cannot be eliminated [21–23].

## Figures and Tables

**Table 1:** Antibiotic susceptibility of *Klebsiella pneumoniae* subspecies pneumoniae (Amp-C beta-lactamase producer).

*Klebsiella pneumoniae* subspecies pneumoniae (Amp-C beta-lactamase producer)	
Drug	MIC interpretation
Amoxicillin/clavulanate	Resistant
Ampicillin	Resistant
Cefuroxime	Resistant
Ceftriaxone	Sensitive
Ertapenem	Sensitive
Gentamicin	Sensitive
Meropenem	Sensitive

## Data Availability

Data is available from the corresponding author on request.
